# What internet- and mobile-based interventions are currently available for adults with overweight or obesity experiencing symptoms of depression? A systematic review

**DOI:** 10.1038/s41366-024-01654-9

**Published:** 2024-10-21

**Authors:** Katja Schladitz, Alina Seibel, Melanie Luppa, Steffi G. Riedel-Heller, Margrit Löbner

**Affiliations:** Institute of Social Medicine, Occupational Health and Public Health (ISAP), Medical Faculty, University of Leipzig, Leipzig, Germany

**Keywords:** Patient education, Risk factors

## Abstract

Given the high prevalence of overweight and obesity and high comorbidity of depressive symptoms, there is a need for low-threshold, accessible care approaches for people with overweight/obesity aimed at improving mental health. Internet and mobile-based interventions (IMI) represent an innovative complementary treatment option. This review systematically searches for IMI aimed at improving mental health in people with overweight/obesity. We conducted a systematic literature search according to the PRISMA (Preferred Reporting Items for Systematic Reviews and Meta-Analyses) criteria in the databases MEDLINE, Cochrane Library, PsycINFO, Web of Science and Google Scholar. Randomized controlled trials (RCTs) of IMI for adults with overweight/obesity and comorbid depressive symptoms aiming at improving mental health were screened and extracted. Study quality was assessed with RoB 2 (revised Cochrane Risk of Bias tool in RCTs). After excluding duplicates, *n* = 790 results were included in title and abstract screening. After full-text-screening of *n* = 26 studies, *n* = 3 RCT studies were included. All interventions aimed to reduce both weight and depressive symptoms. In two RCTs, a significant reduction in both depressive symptoms and weight was achieved. One RCT indicated a significant reduction in depressive symptoms, but not in weight. Two intervention had a duration of 6 months and were guided by health carers, the third takes 3 months and can be used without professional guidance. There is evidence that IMI are effective in improving mental health for people with overweight/obesity and comorbid depressive symptoms. However, currently there are few interventions aiming at reducing depressive symptoms, all targeting English-speaking people. As IMI for depressive symptoms can be easily integrated in the somatic therapy of obesity as additional option and has high public health potential, target group-adapted and low-threshold accessible interventions in different languages should be developed and implemented for improving mental health in people with overweight/obesity. Prospero registration number: CRD42023361771.

## Introduction

According to the World Health Organization (WHO), for adults a body mass index (BMI) of ≥25 is defined as overweight and a BMI of ≥30 as obesity [1]. The prevalence of overweight/obesity has increased worldwide with a steadily rising trend: in 2022, the proportion of people with obesity worldwide has nearly doubled since 1990 [2–4]. Obesity reached pandemic levels and is one the major global public health burdens [1, 3]. Overweight/obesity are caused by multiple factors, including biological, educational, environmental and socioeconomic factors as well as lifestyle behavior [3, 5–7]. Psychological factors also can have a negative impact on weight gain, e.g., affective, anxiety or eating disorders, stigma and discrimination or chronic stress [5, 7, 8]. Excessive weight is associated with a number of adverse health outcomes, e.g., reduced physical health and increased risk of developing chronic diseases, as well as functional impairments [3, 5, 9–11]. It is also associated with reduced mental health [12], e.g., an increased risk of developing depressive disorders [13, 14].

As described, depressive symptoms can be both a cause and a consequence of overweight/obesity. Depressive disorders are also highly prevalent: they are among the most common mental disorders [15, 16], with a steadily rising trend [17, 18]. Likewise, depressive disorders are associated with reduced physical health, increased risk of developing comorbid diseases, more severe course and worse progression of somatic comorbidities and functional impairments [16, 19, 20].

There is evidence for a bi-directional association of overweight/obesity and depression: People with overweight/obesity have an increased risk of developing depressive symptoms [13, 14, 21–25]. Vice versa, having depressive symptoms increases the risk of developing obesity [13, 14, 25–27]. When a person has both overweight/obesity and depression, one condition has a negative impact on the course of the other, as this comorbidity increases the negative impact on physical and mental health and social life, and it is a risk factor for a more severe course of the disease and poorer chances of recovery [13, 22, 28].

When obesity and depression co-occur, both conditions are rarely treated to the same extent and there are little established models for combined treatment [29]. People with severe depressive symptoms are often screened out from weight loss trials, e.g., due to concerns that they may experience aggravation of their depressive symptoms or that they would not be able to achieve similar weight loss like non-depressed participants [30]. Current studies show evidence that integrated interventions that simultaneously address overweight/obesity and depression are promising [31–33]. However, the treatment of overweight/obesity often focuses on the underlying somatic conditions rather than on co-occurring mental health problems [34]. Given the high level of comorbidity, there is a need for low-threshold innovative treatment approaches that address the mental health of people with overweight/obesity.

One of these innovative treatment options are internet- (delivered via web browser on desktop, laptop, tablet computer or smartphone) and mobile-based (delivered via smartphone app) interventions (IMI) in order to give health-related assistance [35–37]. IMI can be accessed at low thresholds, location- and time-independently and at relatively low costs [36, 38, 39]. They can complement healthcare services and bridge waiting time for therapy vacancies, and they can be used in blended-care settings [36, 39, 40]. IMI have the potential to address barriers in healthcare of people with chronic somatic conditions like obesity, e.g., stigmatization, isolation or reduced mobility [41]. Their use requires a number of prerequisites, e.g., technical equipment and the ability to use the internet or trust in data security [36, 39].

The effectiveness of IMI has been demonstrated for a wide range of mental health conditions [36, 39, 42–44], including depression [45, 46], and comparable to conventional “face-to-face” interventions. The user acceptance is predominantly positive [47–49]. IMI are already being integrated into the healthcare system, e.g., in Germany via the possibility of a medical prescription (“Digital Health Applications/DiGA”) [50] or through inclusion in clinical practice guidelines like the “S3 guideline for unipolar depression” [51]. There is also evidence that people with overweight/obesity have positive attitudes and high user acceptance of IMI for depressive symptoms [52, 53].

But research also suggests that adaptation to the target group is crucial as individuals would benefit more from IMI if these interventions were adequately tailored to their specific needs [54–56].

## Objectives

So far, there has been no systematic evaluation of IMI for comorbid depressive symptoms in people with overweight/obesity. There are some reviews that systematically reviewed collaborative approaches to simultaneously treat overweight/obesity and depression, but they either did not focus on psychological interventions or IMI [32, 33] or exclusively searched for mobile interventions [57]. The authors did not consider approaches that specifically target depressive symptoms in people with overweight/obesity [32, 33, 57]. Assessing the effectiveness and quality of IMI targeting depressive symptoms in people with overweight/obesity could help to improve their health care and to establish IMI as a feasible treatment option.

Against this background, the present review aims at the following research questions:

- Are IMI for adults with overweight/obesity who experience comorbid depressive symptoms effective in reducing depressive symptom severity?
- What is the quality of the available evidence of IMI for adults with overweight/obesity and comorbid depressive symptoms?

## Methods

### Registration, protocol and guidelines

The methods, eligibility criteria, strategy for data research and analyses are described in the review protocol [58]. The systematic review was registered in the international prospective register of systematic reviews PROSPERO (CRD42023361771). We systematically evaluated and synthesized data to examine the effectiveness of these IMI according to the 24-step guide by Muka et al. [59] and followed the preferred reporting items for systematic reviews and meta-analyses (PRISMA)-statement for systematic reviews [60].

### Eligibility criteria

We followed the “Population—Intervention—Comparison—Outcome” (PICO) scheme and searched for randomized controlled trials (RCTs) including adults aged 18 years and older with overweight/obesity (BMI ≥ 25) and comorbid depressive symptoms applying IMI.

We defined IMI as clinical interventions targeting depressive symptoms via online-, internet-, web- or mobile-based settings and based on psychological principles according to Kampling et al. [61] (CBT, psychodynamic psychotherapy, behavior therapy or behavior modification, systemic therapy, third wave CBT, humanistic therapies, integrative therapies or other psychological-oriented interventions).

Studies were excluded if the intervention (1) aimed at increasing quality of life or overall well-being without targeting depressive symptoms, (2) did not address mental health directly, but only through changes in weight, or (3) addressed a highly specific population (e.g., pregnant women, individuals after bariatric operations or with a specific somatic disease). Studies were also excluded if the IMI were not compared with one of the following control conditions: treatment as usual (TAU, referring to psychotherapy, pharmacotherapy or both), waiting list, low-level support, psychosocial support, attention placebo or psychological placebo. We only covered articles written in English or German.

To be eligible for review, studies had to include individuals with overweight/obesity who experienced at least mild depressive symptoms, whereas any form of assessment of severity of depressive symptoms was required as primary or secondary outcome.

### Search strategy and study selection

A database search was conducted independently between July 2023 and March 2024 by two researchers (KS and AS) using a comprehensive search strategy for MEDLINE (via PubMed Interface), Cochrane Library, PsycINFO, Web of Science, and Google Scholar (for gray literature) (see Supplementary File 1 for MEDLINE example of search term).

In addition, reference lists of all included studies and relevant systematic reviews were searched manually to identify further potentially relevant articles, and a gray literature search for unpublished studies was conducted using Google and Google Scholar with the search terms.

Two researchers (KS and AS) screened independently first titles and abstracts and then full texts of all search results for duplicates and for eligibility criteria: (1) RCT study design, (2) published in English or German, (3) participants aged ≥18 years with overweight/obesity, and (4) an IMI designed for improving depressive symptoms and based on psychological principles was evaluated. Discrepancies were resolved by discussion between both reviewers and with a third senior researcher (MLö).

### Data extraction

A standardized data extraction form was developed and tested using a random subsample of studies. Two researchers (KS and AS) then independently extracted data from each included study. Discrepancies were resolved by discussion between both reviewers and with a third senior researcher (MLö). The following data were extracted (see Supplementary File 2):

1. Study identification items.
2. Study design characteristics.
3. Participant characteristics.
4. Methodological factors.
5. Outcome data.

### Quality assessment

We used the revised Cochrane Risk of Bias tool in RCTs (RoB 2) [62]. Two researchers (KS and AS) independently assessed the methodological quality of the included studies in the following domains:

1. Bias arising from the randomization process.
2. Bias due to deviations from intended interventions.
3. Bias due to missing outcome data.
4. Bias in measurement of the outcome.
5. Bias in selection of the reported result.

All studies were assigned to the three corresponding overall risk-of-bias-grades following [62]: *high, low* or *some concerns* in each domain and an overall rating was derived. Discrepancies were resolved by discussion between both reviewers and with a third senior researcher (MLö).

## Results

### Study selection

**Fig. 1:**
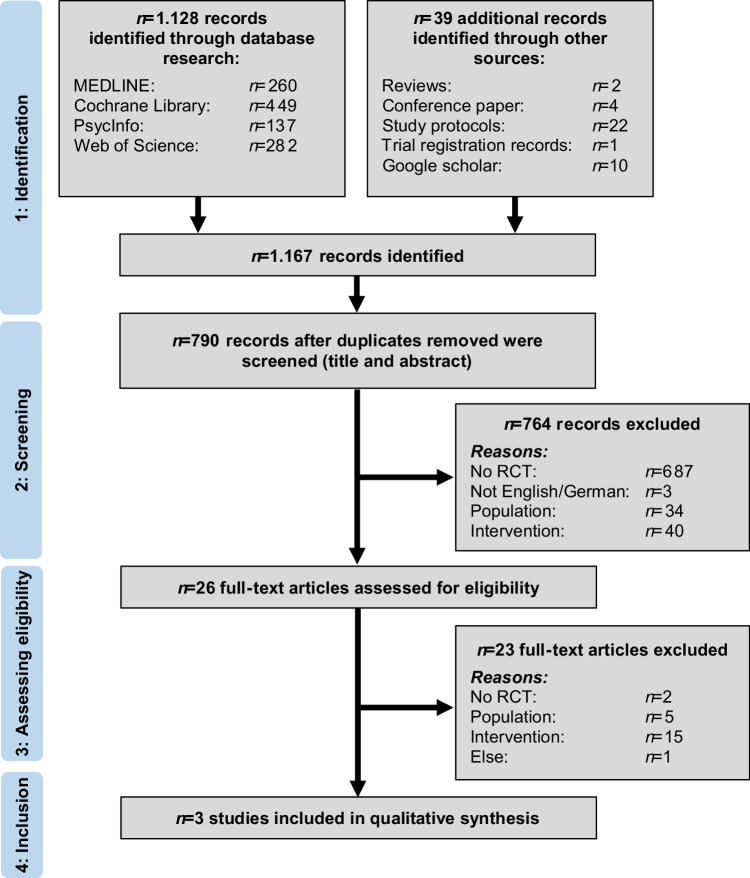
Study selection process-flow diagram. RCT Randomized controlled trial.

The selection process is illustrated in Fig. 1. A total of 1167 records could be identified through literature research in databases and gray literature, 32.3% (*n* = 377) were removed as duplicates. After screening titles and abstract of the remaining *n* = 790 studies, 96.7% (*n* = 687) were removed mostly because they were no RCTs (e.g., trial registration records, observational studies or reviews). Of the *n* = 26 articles included in full text assessment, three studies met all eligible criteria and were included in the review. At this stage, most of the interventions had to be removed because they did not address depressive symptoms directly (only mediated via weight loss) or they were no clinical psychological interventions. A meta-analysis could not be performed due to the small number of identified studies.

### Description of selected studies

The three studies by Ma et al. [63], Lv et al. [64] and Young et al. [65], published between 2019 and 2023, covered two distinct interventions: *Integrated Coaching for Better Mood and Weight (I-CARE)* in study 1 [63] respectively the updated version *I-CARE 2* in study 2 [64] and *Self-Help, Exercise and Diet using Information Technology (SHED-IT) Recharge* in study 3 [65]. These interventions differed in duration: study 1 and 2 (I-CARE/I-CARE 2) applied an intervention over 6 months (study 1 also included a subsequent 6-months phase for maintenance) and study 3 (SHED-IT Recharge program) covered 3 months. While study 3 was completely self-guided and did not include medical or therapeutical feedback, study 1 and 2 were guided interventions with nine face-to-face-sessions and monthly telephone sessions during the maintenance phase (only study 1).

Study 1 and 2 [63, 64] were conducted in the USA (California), study 3 [65] in Australia (Newcastle and Hunter region). The target population of study 1 and 2 were adults of all gender with obesity (BMI ≥ 30 respectively ≥27 for Asian adults) and at least moderate depressive symptoms (Patient health questionnaire (PHQ-9) ≥ 10). Study 3 targeted male adults with overweight or obesity (BMI ≥ 25 and ≤42) that had at least mild depressive symptoms (PHQ-9 ≥ 5). Study 1 and 2 included predominantly female participants (70% resp. 76%). The population of study 3 included men who were 29% Australians with overweight and 71% with obesity, 58% had mild and 42% moderate-to-severe depressive symptoms according to PHQ-9. The sample size varied between *N* = 106 [64], *N* = 125 [65] and *N* = 409 [63]. Table 1 shows details of the study samples.

**Table 1:** Descriptions of study samples.

Authors, year, country	Number of participants: Total (IG)	Eligibility criteria	Exclusion criteria	Age (in years): Mean ± SD	Gender distribution	Depression: scale Mean ± SD, mild/moderate/severe symptoms %	BMI, weight: Mean ± SD, % Overweight/Obesity
Ma et al. (2019) [63], USA	409 (204)	Age ≥18, BMI ≥ 30 (≥27 for Asian adults), PHQ-9 ≥ 10	Inability to speak or read English, moved or leaving the health system, refused to participate, comorbidities that prohibited participation, substance or drug use, psychiatric care outside the health system, pregnant or lactating	51 ± 12.1	70% female	PHQ-9: 13.8 ± 3.1SCL-20: 1.5 ± 0.5	BMI 36.7 ± 6.4
Lv et al. (2023) [64], USA	106 (71)	Age ≥18, BMI ≥ 30 (≥27 for Asian adults), PHQ-9 ≥ 10	Serious medical or psychiatric comorbidities (cardiovascular disease, active suicidal ideation/plan, active alcohol/substance) not fluent in English, transferred or psychiatric care outside of the health system, moved/moving out of area, functional magnetic resonance imaging (fMRI) exclusion or fMRI intolerant/decline reschedule, pregnant/lactating, active bulimia nervosa, bariatric surgery	47 ± 11.9	76% female	PHQ-9: 12.8 ± 2.8 SCL-20: 1.2 ± 0.7	BMI 37.1 ± 6.0
Young et al. (2021) [65], Australia	125 (62)	Male gender, Age 18–70 years, BMI ≥ 25.0 and ≤42.0, PHQ-9 score ≥5	Serious risk of suicide, new antidepressant medication or changed dose (past 4 weeks), started/changed psychotherapy (past 4 weeks), bariatric surgery (past 12 months) or planned to have during the study, unable to speak, read or understand English, planned to move out of the area during the study period, participating or planned to participate in a concurrent weight loss program, not willing to be randomized, lost 5% or more of body weight (past 6 months), did not provide a doctor’s clearance if risks were identified on pre-exercise screener	48.4 ± 11.7	100% male	PHQ-9: 9.2 ± 4.1 58% mild, 42% moderate-to-severe depressive symptoms	Weight 103.8 ± 15.8 kg 29% overweight, 71% obesity

*IG* Intervention Group, *SD* standard deviation, *BMI* body mass index, *PHQ-9* Patient Health Questionnaire, *SCL-20* Depression Symptom Checklist.

All studies were RCTs with one intervention and one control group: study 1 and 2 by Ma et al. [63] and Lv et al. [64] compared the digital intervention to Treatment-as-Usual (TAU), while study 3 by Young et al. [65] compared to waitlist. All studies randomized participants using different methods: the covariate adaptive minimization method of Pocock and Simon [66] in study 1 [63], a 2:1 covariate-adaptive minimization method [67] in study 2 [64], and stratification by antidepressant medication status, depression severity and BMI in the third study [65]. Study 3 reported that the intervention and control groups were comparable in sociodemographic and psychometric characteristics in both conditions, whereas study 1 and 2 did not provide explicit information on comparability. Table 2 shows information on study characteristics.

**Table 2:** Characteristics of the included studies.

Source	Treatment condition	Assessments	Primary outcomes measurements	Secondary mental health outcomes measurements	Summary of effects on depressive symptoms (primary outcome)	Summary of effects on weight (secondary outcome)
Ma et al. (2019) [63]	Exposure vs. TAU control	6 months (intensive treatment phase, mid-intervention), 12 months (maintenance phase, post-intervention)	SCL-20 BMI	GADS (Anxiety)	6 months (mid-intervention): Significant treatment effect (between-group SCL-20 mean difference = −0.3, 95% CI [-0.4, -0.1], *p* < 0.001). 12 months (post-intervention): Significant treatment effect (between-group SCL-20 mean difference = −0.2, 95% CI [−0.4, 0], *p* = 0.01, *d* = 0.23).	6 months (mid-intervention): Significant treatment effect (between-group BMI mean difference = −0.6, 95% CI [−0.9, −0.3]; *p* < 0.001). 12 months (post-intervention): Significant treatment effect (between-group BMI mean difference = −0.7, 95% CI [−1.1, −0.2]; *p* = 0.01, *d* = 0.28).
Lv et al. (2023) [64]	Exposure vs. TAU control	6 months (post-intervention) + 2-month-mid-intervention assessment	SCL-20 BMI	GADS (Anxiety)	6 months (post-intervention): Significant treatment effect (between-group SCL-20 mean difference = −0.3, 95% CI [−0.6, –0.1], *p* = 0.002).	6 months (post-intervention): No significant treatment effect (between-group BMI mean difference = −0.3, 95% CI [−1.0, 0.4]; *p* = 0.45).
Young et al. (2021) [65]	Exposure vs. wait list control	3 months (post-intervention), 6 months (follow up)	PHQ-9 Weight loss (measured)	BDI (depression) MDRS-22 (male depression) GADS (Anxiety)	3 months (post-intervention): Significant treatment effect (adjusted PHQ-9 mean difference = −2.4, 95% CI [−3.9, −0.8], *p* < 0.01, *d* = 0.55) 6 months (follow-up): Significant treatment effect (adjusted weight mean difference = −2.4, 95% CI [−4.0, −0.7], *p* < 0.01, *d* = 0.52).	3 months (post-intervention): Significant treatment effect (adjusted weight mean difference = −3.1 kg, 95% CI [−4.3, −1.9], *p* < 0.001, *d* = 0.9, and adjusted BMI mean difference = −1.0, 95% CI [−1.3, −0.6], *p* < 0.001, *d* = 0.9) 6 months (follow-up): Significant treatment effect (adjusted weight mean difference = −3.6 kg, 95% CI [−5.1, −2.0], *p* < 0.001, *d* = 0.8, and adjusted BMI mean difference = −1.1, 95% CI [−1.6, −0.6], *p* < 0.001, *d* = 0.8).

*TAU* treatment as usual, *CI* confidence interval, *d* Cohen’s *d*, *BMI* body mass index, *SCL-20* Depression Symptom Checklist, *BDI* Beck Depression Inventory, *GADS* Generalized Anxiety Disorder scale, *PHQ* Patient Health Questionnaire, *MDRS-22* Male Depression Risk Scale.

All studies applied individual interventions both based on social cognitive theory [68] and cognitive behavioral therapy, including problem solving, goal setting and behavioral activation. All interventions aimed at losing weight as well as reducing depressive symptoms and therefore comprised dietary and physical activity components and mental health components. Study 1 and 2 [63, 64] integrated and adapted two existing interventions to one synergistic curriculum (I-CARE/I-CARE-2): *the Group Lifestyle Balance (GLB)* [69] program for weight loss as *I-CARE-Lifestyle* component and the *Program to Encourage Active, Rewarding Lives for Seniors (PEARLS)* [70] for improving mental health *as I-CARE-Mood Session*-component. The intervention I-CARE/I-CARE-2 includes a significant amount of face-to-face-sessions and therefore must be regarded as hybrid and guided and not as internet-based-only intervention. Study 3 by Young et al. [65] applied the unguided gender-tailored *SHED-IT Recharge Program*, an adaption of the *SHED IT-Weight Loss Program for Men* [71]. It combines printed (hand- and logbook) and online (videos and interactive modules) resources and devices for self-monitoring (pedometer), but no face-to-face-session or feedback by a person. Additionally, both programs provide a *MyFitnessPal*-Account for tracking and self-monitoring physical activity and dietary intake. Table 3 shows details of the interventions.

**Table 3:** Characteristics of the intervention.

Source	Name of intervention & trial	Duration	Therapeutic approaches	Intervention components or short description	Mental health components (focusing on reducing depressive symptoms)	Weight loss/dietary components (focusing on reducing weight)	Guidance/ therapeutical feedback	Elements
Ma et al. (2019) [63]	*Name of intervention:* • I-CARE (Integrated Coaching for Better Mood and Weight) *Name of trial*: • RAINBOW (Research Aimed at Improving Both Mood and Weight)	12 months (6 months intensive treatment phase + 6 months maintenance phase	Social cognitive theory (Bandura, 2004), cognitive behavioral therapy	*Synergistic 2-component curriculum (I-CARE):* 1. Group Lifestyle Balance (GLB) program—somatic focus on obesity 2. Program to Encourage Active, Rewarding Lives for Seniors (PEARLS)—mental health focus based on problem-solving therapy *2 phases:* • Intensive treatment • Maintenance	Problem solving and goal setting: identify a problem • Set a goal • Brainstorm solutions • Choose a solution • Develop, implement and evaluate an action plan [72]	• Problem solving • Goal setting and self-monitoring • Healthy meal plan • Mindful eating • Stress and time management • Physical activity plan • Social support	Guided	*Intensive treatment phase:* 9 face-to-face sessions (90 min) • 11 home-viewed GLB videos (20–30 min) • Stepwise increases in dose and number of antidepressant medications *Maintenance phase:* • Telephone sessions (monthly, 15–30 min) • Self-care materials (handouts, DVD set/online access for GLB videos) • Activity tracker • MyFitnessPal-Account
Lv et al. (2023) [64]	*Name of intervention:* • I-CARE2 (Integrated Coaching for Better Mood and Weight) *Name of trial:* • ENGAGE-2 (Engaging self-regulation targets to understand the mechanisms of behavior change and improve mood and weight outcomes)	6 months (intensive treatment phase), no maintenance phase	Social cognitive theory (Bandura, 2004), cognitive behavioral therapy	*Synergistic 2-component curriculum (I-CARE2):* 1. Group Lifestyle Balance (GLB) program—somatic focus on obesity 2. Program to Encourage Active, Rewarding Lives for Seniors (PEARLS)—mental health focus based on problem-solving therapy	Not described	*Not described in detail, only mentioned:* • Problem-solving • Behavioral activation	Guided	• 6 face-to-face sessions in the first 2 months • 3 additional face-to-face sessions • 11 home-viewed GLB videos over the next 4 months • Self-monitoring of weight and diet • Activity tracker & Fitbit application • Antidepressant medications
Young et al. (2021) [65]	*Name of intervention & trial:* • SHED-IT (Self-Help, Exercise and Diet using Information Technology): Recharge-Intervention &-Trial	3 months	Social Cognitive Theory (Bandura, 2004), cognitive behavioral therapy	Male gender-tailored eHealth weight loss program that teaches men how to lose weight through sustainable and realistic lifestyle behavior change	*4 modules:* • Cognitive restructuring • Mindfulness and relaxation • Behavioral activation • Relapse prevention *Targeting behavior change techniques of* • Reducing negative emotions • Problem solving • Reframing • Self talk	*Lifestyle and psychoeducation:* • Men’s health • Weight loss • Goal setting and self-monitoring • Evidence based tips to increase or reduce key behaviors linked to physical and mental health (e.g., physical activity, sleep, alcohol, unhealthy drinks and nutrition).	Self-guided, no feedback	• Handbook (printed) • Logbook with weekly tasks (printed) • Videos (online) • Interactive mental fitness modules (online) • MyFitnessPal-Account (online) • Text Messages (Phone) • Pedometer (device)

### Quality assessment

**Fig. 2:**
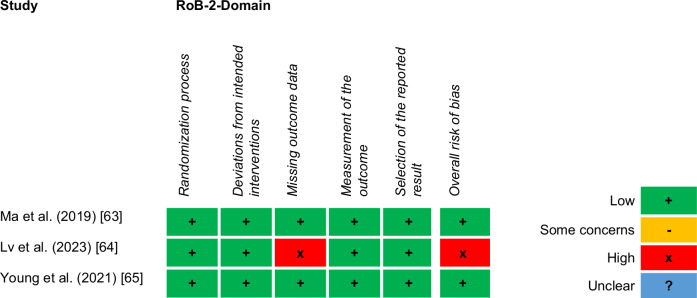
Risk of bias in included randomized controlled trials according to the revised Cochrane Risk of Bias tool in RCTs (RoB 2).

Risk of bias was assessed independently by two reviewers (KS and AS) using the Cochrane Collaboration’s Risk of Bias 2 (RoB-2) tool [62]. For each of the five domains, the risk of bias was estimated, and an overall rating was derived. Two studies [63, 65] had a low risk of bias in all domains and therefore a low overall risk of bias. One study—a pilot study and therefore without predefined analysis plan reported in a trial protocol—had a high risk of bias because information on the percentage of missing outcome data was not reported and analyzed as a potential source of bias [64]. Figure 2 shows the results of the domain and overall ratings (the detailed risk of bias assessment can be found in Supplementary File 3).

### Findings of included studies

In all three RCTs [63–65], the reduction of depressive symptoms was significantly greater in the intervention group than in the control group.

Ma et al. [63] reported a significant treatment effect with an adjusted between-group mean difference in depressive symptoms (Symptom check list (SCL-20)-score) of −0.2 (*p* = 0.01) and small effect size (*d* = 0.23) (post-intervention, 12 months). Lv et al. [64] reported a significant treatment effect with a adjusted between-group mean difference in depressive symptoms (SCL-20-score) of −0.3 (*p* = 0.002) (post-intervention) (no effect size reported). As there was no follow-up-assessments, long-term-maintenance could not be analyzed.

In study 3 [65], participants receiving the 3-month-intervention had a significantly higher reduction in depressive symptoms in three different validated scores:

- Patient Health Questionnaire (PHQ-9, primary outcome): adjusted between-group mean difference of −2.4 (*p* < 0.01), representing a medium effect size (*d* = 0.55). These improvements were maintained at 6 months, with an adjusted between-group mean difference of −2.4 (*p* < 0.01) and medium effect size (*d* = 0.52).
- Beck Depressive Symptoms (BDI) (secondary outcome): adjusted between-group mean difference of −4.5 (*p* < 0.001) and medium effect size (*d* = 0.71). This effect was reduced at 6 months, with an adjusted between-group mean difference of −2.4 (*p* < 0.001) and small effect size (*d* = 0.32).
- Male depression risk scale (MDRS-22) (secondary outcome): adjusted between-group mean difference of −7.1 (*p* = 0.02) and small effect size (*d* = 0.43). These improvements were not significant anymore at 6 months, with an adjusted between-group mean difference of −5.6 (*p* = 0.06) and small effect size (*d* = 0.34).

Concerning weight loss, two of three studies reported a significant effect on weight: Ma et al. [63] reported an adjusted between-group mean difference in BMI of −0.7 (*p* = 0.01) with small effect size (*d* = 0.28). Young et al. [65] also reported a significant higher reduction of weight with an adjusted between-group mean difference of −3.1 kg (*p* < 0.001) representing a large effect size (*d* = 0.92), and BMI, adjusted between-group mean difference of −1.0 (*p* < 0.001) with large effect size (*d* = 0.95). This improvement had slightly decreased at 6 months, with an adjusted between-group mean difference in weight of −3.6 kg (*p* < 0.001) and large effect size (*d* = 0.84), and in BMI, adjusted between-group mean difference of −1.1 (*p* < 0.001) and large effect size (*d* = 0.82).

## Discussion

### Principal findings

The aim of this review was to systematically search for RCTs of IMI that reduce depressive symptoms in adults with overweight/obesity. We identified no trial that aimed at reducing depressive symptoms without simultaneously aiming at weight loss. We also identified two interventions (within three studies) that aimed to reduce depressive symptoms as well as weight: the I-CARE/I-CARE 2 program [63, 64] and the SHED-IT-Recharge program [65]. All trials reported a reduction in depression scores with small to moderate effect sizes and both reported a reduction in weight/BMI with large effect sizes. Two studies are of high methodological rigor [63, 65], and one study was a pilot study. The I-CARE and I-CARE 2 as well as the SHED-IT Recharge program combined a weight loss and mental health program in one synergistic, intertwined approach, and both were based on CBT. Both interventions were guided IMI and were combined with other components (“blended”): The I-CARE/I-CARE 2 intervention also integrated face-to-face sessions and an adaption of antidepressant medication, and the SHED-IT Recharge trial also used printed materials. Besides, both integrated technological devices to increase physical activity (pedometer). It is therefore difficult to estimate the individual effect of the IMI components as they are integrated into complex approaches. The I-CARE/I-CARE 2 intervention in particular includes a significant proportion of face-to-face sessions which is not fully consistent with the IMI definition.

The interventions differ in important aspects. The SHED-IT Recharge program [65] is a short-term intervention of 3 months, self-guided with no therapeutical feedback or face-to-face elements. The I-CARE/I-CARE 2 program [63, 64] is a 6-month long-term intervention, with the option to add 6 months of maintenance components [63], guided and with a significant amount of face-to-face sessions (and telephone sessions in the maintenance phase). The SHED-IT Recharge program is designed specifically for men and is based on the symptoms of male depression, while the I-CARE/I-Care 2 program is gender neutral. Therefore, they are difficult to compare and to generalize. This may illustrate the variety of ways in which both overweight/obesity and comorbid depression can be addressed within an IMI approach.

Mediating variables or mechanisms of change for reduction of depressive symptoms were analyzed for both programs. Association with sleep-related variables such as sleep quality [72], sleep disturbance and sleep-related impairment [73] were found, which is supported by empirical findings [74–77]. Drew et al. [72] also identified behavioral activation as a mediator for depressive symptom reduction, which is an important element of CBT therapy for depression and consistent with research [78, 79]. They also identified some weight management-related mediators, e.g., at least moderate physical activity and avoidance of unfavorable foods (energy-dense and nutrient-poor) which is also consistent with research [80–82]. To prevent or treat depressive symptoms in people with overweight/obesity, promotion of at least moderate physical activity, sleep hygiene, healthy dietary habits, cognitive flexibility and behavioral activation should therefore be integrated into mental health IMI for this target group [72, 73]. This should apply whether they use a collaborative or depressive symptom-focused approach.

### Three approaches of IMI for people with overweight/obesity and comorbid depressive symptoms

There are three clusters of IMI that we know about from researching and screening. The first intend to address both the goals of reducing weight and depressive symptoms in a collaborative approach, the second focus on mental health but do not target people with overweight/obesity, and the third cluster include CBT elements aimed at weight loss but do not explicitly address mental health.

#### Collaborative approaches

The authors of two studies [63, 65] concluded that collaborative approaches are promising and that it would be reasonable to combine the aims of reducing both weight and depressive symptoms. Mechanisms of change like goal setting, cognitive restructuring, problem solving or mindfulness can be applied for keeping dietary restrictions and increasing physical activity as well as for the treatment of depressive symptoms. There seem to be synergy effects in using the same change mechanisms for both goals at the same time.

Drew et al. [72] identified two somatic mediators for depressive symptom reduction, which are also integral elements in weight loss interventions: an increase in moderate-to-vigorous activity and a reduction of intaking energy-dense nutrient-poor food. There is broad evidence that physical activity can lead to a reduction in depressive symptoms and an increase in mental wellbeing of patients with depressive disorders, as well as to weight loss [80, 81, 83]. Kris-Etherton et al. [82] emphasize that healthy eating patterns based on food-based dietary recommendations and nutrient requirements can be beneficial in preventing and treating depression. Swainson et al. [84] identify a positive impact of dietary interventions in the treatment of depressed patients. Some forms of diets that meet current nutritional recommendations (such as Mediterranean or Norwegian) are associated with a lower risk of developing depressive symptoms [85–87]. Additionally, emotional eating often occurs as depressive symptom. It can be used as a strategy to cope with negative emotions and can mediate between depression and overweight/obesity [88, 89]. This eating pattern is characterized by a preference for energy-dense nutrient-poor foods, regardless of hunger signals or biological needs and can contribute to weight gain [88]. Devenport et al. [90] summarize that depressive feelings consistently elicit eating behaviors that are not consistent with health-promoting nutritional recommendations. Collaborative approaches can combine promotion of emotions regulation skills and beneficial coping mechanisms with education about healthy diets and eating behavior.

However, the interventions [63–65] also show that collaborative approaches are demanding and time-consuming for users and require a high level of commitment. This also applies to non-digital collaborative approaches. It is a consequence from the complexity and scope of the content that needs to be taught with regard to depression and overweight/obesity treatment [32, 91–93]. Such complex interventions can be overwhelming and discouraging for some users due to time or capacity reasons. The use of collaborative programs requires a lot of energy and drive—both of which can be reduced as comorbid depressive symptoms. If the impairment caused by depressive symptoms is severe and does not allow to use a complex collaborative program, it may be appropriate to sequentially target the depressive symptoms first.

#### Approaches focusing on mental health

IMI in this groups aim to reduce depressive symptoms and to improve mental health only. We also found IMI in the study selection process, e.g., Heriseanu et al. [94]. The effectiveness of such IMI in the general population is well documented [39, 40, 43, 45, 46]. These may include general lifestyle changes such as sleep hygiene, physical activity and favorable dietary habits which are associated with better mental health, but they do not aim at weight management. These IMI can be used by people with overweight/obesity, e.g., in addition to weight management provided by a general practitioner or specific weight loss programs. And they can also be used as stand-alone support option when weight loss is not desired or indicated.

However, people with overweight/obesity and comorbid depressive symptoms wish for target-group specific IMI that combine the topics of weight management and depression and include topics like physical activity, nutrition, coping skills and adaption to new life situations and social interaction [52, 56, 95]. These interventions should address specific issues arising from the comorbidity, such as self-stigma, negative body image, reduced physical activity and functional impairment [96–99]. Therefore, there is a need for adapted online interventions to reduce depressive symptoms in people with overweight/obesity without simultaneously targeting weight. But we could not find any specific programs for this target group in this systematic review. But research in this field is ongoing and first interventions are developed and evaluated [100].

#### Approaches that include CBT elements for weight loss purposes

Within the screening process, we found some studies that aimed at weight loss and integrated CBT elements for supporting weight management. They integrated mostly goal setting and motivation theory, psychoeducation, examining and overcoming destructive thoughts and barriers related to dietary restrictions and physical activity [101–104]. This type of studies did not explicitly address people with depressive symptoms. Trial participants were usually a mix of people that are non-depressed, mildly depressed and moderately depressed (severe symptom burden was usually formulated as an exclusion criterion), and the primary aim was to improve mental health through weight loss. Some studies addressed bariatric surgery candidates during their waiting time, comparing whether the existing program for preparation for surgery, which takes place in presence, is just as effective web-based [105, 106].

While these trials were generally successful in achieving weight loss, the results for mental health were mixed. Some studies failed to show significant improvements in depressive symptoms [101, 103], while others showed significant reductions on depressive symptoms compared with TAU or control groups [102, 104]. These findings highlight the importance of explicitly addressing depressive symptoms and developing (1) collaborative weight management and mental health IMI and (2) tailored mental health IMI for people with overweight/obesity. It cannot be assumed that depressive symptoms will automatically improve if weight is reduced.

### Why do we need IMI that address depressive symptoms without simultaneous weight management that are adapted to the target group of people with overweight/obesity?

Obesity is often treated as a “more urgent” problem because of its potentially serious somatic consequences. Depressive symptoms are often only perceived as a consequence of overweight/obesity, that would decrease ‘automatically’ once the weight is reduced [34]. Current study results on online weight reduction programs (without a specific depression intervention) show that there are positive effects on the improvement of depressive symptoms in people with overweight/obesity [102, 107, 108].

But depressive symptoms can also occur additionally to overweight/obesity and, as described in the introduction-section, the relationship is bi-directional [13, 109]. That means depressive symptoms can be caused or aggravated by overweight/obesity, and they can also lead to weight gain. Treatment of depressive symptoms should therefore not be postponed after overweight/obesity-treatment, but should be offered at the same time or even first, depending on medical urgency and preference of the patients. It is known that there is a risk of depressive symptoms worsening, becoming chronic and becoming more severe if they are not treated early [16, 61]. The use of IMI can help to provide psychological support to people with overweight/obesity in order to reduce their depressive symptoms at an early stage and to prevent a worsening and chronification of depressive symptoms.

There is a demand from the target group for customized mental health IMI [56, 95]. In addition, such IMI have several advantages for people with chronic somatic diseases and physical impairments [110, 111], e.g., independence of time and location, accessibility in regions with structural health care gaps, anonymous use and a reduced risk of stigmatization. They can be a component in stepped care and blended-care approaches and while waiting for treatment appointments. They can be used as complementary component in somatic care. And if a person is reluctant to start psychotherapy and may be skeptical about psychotherapeutic approaches, mental health IMI can help to increase their openness and willingness to start a psychotherapy [110, 111]. IMI can sustainably improve the health care situation of people with overweight/obesity and comorbid depressive symptoms. Their use enables various healthcare providers in different care settings to provide their patients with easy and quick access. Accordingly, IMI can be seen as a valuable additional treatment option as part of a stepped care approach for people with overweight/obesity.

### Strengths and limitations

This systematic review provides the first overview of current evidence of IMI that reduce depressive symptoms in adults with overweight/obesity based on psychological techniques.

Certain limitations need to be mentioned. First of all, this review is based on only three studies covering two interventions. This shows the urgent need to develop IMI that aims at reducing depressive symptoms in people with overweight/obesity. Both interventions are complex because they combine the two goals weight loss and depressive symptoms reduction in a collaborative approach. This requires a lot of effort and is time consuming for the users. Second, these studies are heterogeneous and their interventions differ in important characteristics according to Lin & Baumeister [35] (e.g., guided and unguided, hybrid or stand-alone). They are also different in length (3–6 months) and in the target group (one study aims only at men). A generalization of their effectiveness on the general population cannot be derived.

Therefore, this review highlights the need to develop and evaluate targeted low-threshold interventions for people with overweight/obesity aimed at reducing their depressive symptoms and to improve their mental wellbeing.

## Conclusions

Our review shows the potential of IMI to reduce depressive symptoms in people with overweight/obesity. There is evidence that these IMI are effective in improving the mental health of people with overweight/obesity and comorbid depressive symptoms. However, currently few interventions for this target group that aim to reduce depressive symptoms are available, and they target English-speaking people. More studies with high quality study designs (RCTs) and interventions in different languages (e.g., German) are needed.

The interventions identified in this review integrate two aims of reducing weight as well as depressive symptoms. IMI that focus on depressive symptoms can be easily adopted in the somatic therapy of obesity as additional tool, e.g., by general practitioners, nutritionists or sport therapists. Therefore, we need more variety in the interventions for the target group of people with overweight/obesity. We need IMI that combine weight and mental health goals as well as IMI that focus mainly or entirely on mental health. IMI with low support or completely unguided would be important to integrate them into everyday life and treatment. Different target groups should be addressed, e.g., older people or people with need for easy language.

Given the potential of IMI to prevent and treat depressive symptoms in a low-threshold and low-cost manner, further interventions should be developed, evaluated, and implemented that are targeted and accessible to this population.

## Supplementary information

Example for search strategy

Data Extraction Form

RoB_2.0 Assessments
