# Supplementary material for: What internet- and mobile-based interventions are currently available for adults with overweight or obesity experiencing symptoms of depression? A systematic review
Source: Int J Obes (Lond). 2024 Oct 21;49(1):63–75. doi: 10.1038/s41366-024-01654-9 (PMC11683006; doi:10.1038/s41366-024-01654-9)
Supplement: Supplementary file 1 — Example for search strategy [file 41366_2024_1654_MOESM1_ESM.docx]

**Example for search strategy:**

**MEDLINE via PubMed**

(((obesity[Title/Abstract] OR overweight[Title/Abstract] OR adiposity[Title/Abstract] OR "metabolic syndrome"[Title/Abstract] OR "body mass index"[Title/Abstract])

AND

(depress*[Title/Abstract]) )

AND

((internet[Title/Abstract] OR online[Title/Abstract] OR web[Title/Abstract] OR computer[Title/Abstract] OR mobile[Title/Abstract] OR app[Title/Abstract] OR smartphone[Title/Abstract] OR m-health[Title/Abstract] OR "mobile health"[Title/Abstract] OR "e-health"[Title/Abstract] OR "e-mental health"[Title/Abstract] OR iCBT[Title/Abstract] OR cCBT[Title/Abstract] OR IMI[Title/Abstract])))

AND

((intervention[Title/Abstract] OR psychotherapy[Title/Abstract] OR therapy[Title/Abstract] OR "cognitive behavioral therapy"[Title/Abstract] OR cbt[Title/Abstract]))
