# Supplementary material for: What internet- and mobile-based interventions are currently available for adults with overweight or obesity experiencing symptoms of depression? A systematic review
Source: Int J Obes (Lond). 2024 Oct 21;49(1):63–75. doi: 10.1038/s41366-024-01654-9 (PMC11683006; doi:10.1038/s41366-024-01654-9)
Supplement: Supplementary file 3 — RoB_2.0 Assessments [file 41366_2024_1654_MOESM3_ESM.docx]

Revised Cochrane risk-of-bias tool for randomized trials (RoB 2)

Edited by Julian PT Higgins, Jelena Savović, Matthew J Page, Jonathan AC Sterne
on behalf of the RoB2 Development Group

**Version of 22 August 2019**

The development of the RoB 2 tool was supported by the MRC Network of Hubs for Trials Methodology Research (MR/L004933/2- N61), with the support of the host MRC ConDuCT-II Hub (Collaboration and innovation for Difficult and Complex randomised controlled Trials In Invasive procedures - MR/K025643/1), by MRC research grant MR/M025209/1, and by a grant from The Cochrane Collaboration.


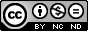


This work is licensed under a [Creative Commons Attribution-NonCommercial-NoDerivatives 4.0 International License](http://creativecommons.org/licenses/by-nc-nd/4.0/).

| **Study 1**  **-----------------**  **Study details**   \| **Reference** \| Ma, Jun; Rosas, Lisa Goldman; Lv, Nan; Xiao, Lan; Snowden, Mark B.; Venditti, Elizabeth M. et al. (2019): Effect of Integrated Behavioral Weight Loss Treatment and Problem-Solving Therapy on Body Mass Index and Depressive Symptoms Among Patients With Obesity and Depression: The RAINBOW Randomized Clinical Trial. In: *JAMA* 321 (9), S. 869–879. DOI: 10.1001/jama.2019.0557. \| \| --- \| --- \|   **Study design**   \| X \| Individually-randomized parallel-group trial \| \| --- \| --- \|   **For the purposes of this assessment, the interventions being compared are defined as**   \| Experimental: \| Exposure \| Comparator: \| TAU \| \| --- \| --- \| --- \| --- \|  \| **Specify which outcome is being assessed for risk of bias** \| Depressive symptoms severity (SCL-20) \| \| --- \| --- \|  \| **Specify the numerical result being assessed.** \| For the SCL-20, the mean score declined from 1.5 (SD, 0.5) to 1.1 (SD, 1.0) among participants in the intervention group compared with a change from 1.5 (SD, 0.6) to 1.4 (SD, 1.3) among participants in the usual care group (between-group mean difference,−0.2 [95%CI, −0.4 to0]; *P* = .01). The Cohen d effect was 0.23 (95%CI, −0.03 to 0.34) for SCL-20 score. \| \| --- \| --- \|   **Is the review team’s aim for this result…?**   \| X \| to assess the effect of *assignment to intervention* (the ‘intention-to-treat’ effect) \| \| --- \| --- \|   **Which of the following sources were obtained to help inform the risk-of-bias assessment?**  X Journal article(s) with results of the trial  X Trial protocol |
| --- | --- | --- | --- | --- | --- | --- | --- | --- | --- | --- | --- | --- | --- | --- |

Domain 1: Risk of bias arising from the randomization process

| **Signalling questions** | **Comments** | **Response options** |
| --- | --- | --- |
| **1.1 Was the allocation sequence random?** | “Participants were randomly assigned to receive usual care or the Integrated Coaching for Better Mood and Weight intervention using an online system. The covariate adaptive minimization method of Pocock and Simon was used to achieve better-than-chance marginal balance across the baseline characteristics of clinic, age, sex, race/ethnicity, education level, BMI, SCL-20 score, use of antidepressant medications, and number of hospitalizations during the past year. The minimization algorithm used the range distance measure and equal weight for the 9 balancing factors to calculate imbalance scores. The randomization probabilities applied the biased-coin method (ie, 2/3 to1/3) of Efron to protect allocation concealment. Investigators, the data and safety monitoring board, outcome assessors, and the data analysts were blinded until after completion of the primary data review through 12 months.” (p. 870). | Y |
| **1.2 Was the allocation sequence concealed until participants were enrolled and assigned to interventions?** | Trial Protocol: “A staff person not involved in outcome assessments or data analysis will perform randomization using a web-based system” (p. 4)  Allocation was realized via an online system (= remote administered). | Y |
| **1.3 Did baseline differences between intervention groups suggest a problem with the randomization process?** | “The covariate adaptive minimization method of Pocock and Simon was used to achieve better-than-chance marginal balance across the baseline characteristics of clinic, age, sex, race/ethnicity, education level, BMI, SCL-20 score, use of antidepressant medications, and number of hospitalizations during the past year. The minimization algorithm used the range distance measure and equal weight for the 9 balancing factors to calculate imbalance scores. The randomization probabilities applied the biased-coin method (ie, 2/3 to1/3) of Efron to protect allocation concealment. Investigators, the data and safety monitoring board, outcome assessors, and the data analysts were blinded until after completion of the primary data review through 12 months.” (p. 870).  Trial Protocol: “ Specifically, for each patient about to be randomized, the system automatically calculates an imbalance score for each of the above-mentioned baseline covariates, and then a total imbalance score, S, by summing across the covariates. If S = 0, the randomization probability for receiving the intervention for that patient will be set to ½, and if S < 0 (S > 0), the randomization probability will be set to 2/3 (1/3).” (p. 4)  No statistical tests of potential differences between groups, but a visual inspection of baseline sociodemographic characteristics did not suggest differences and problems with randomization process. The authors used methods to correct potential imbalances using a study weight (“total imbalance score”). | PN |
| **Risk-of-bias judgement** |  | Low |
| Optional: What is the predicted direction of bias arising from the randomization process? |  | NA |

Domain 2: Risk of bias due to deviations from the intended interventions (effect of *assignment to intervention*)

| **Signalling questions** | **Comments** | **Response options** |
| --- | --- | --- |
| **2.1. Were participants aware of their assigned intervention during the trial?** | Trial Protocol: “By design, treatment assignments are identifiable to participants and the lifestyle coach (…) (p. 4)  No blinding possible because of intervention design | Y |
| **2.2. Were carers and people delivering the interventions aware of participants' assigned intervention during the trial?** |  | Y |
| **2.3. If Y/PY/NI to 2.1 or 2.2: Were there deviations from the intended intervention that arose because of the trial context?** | (Serious) adverse events and adherence are consistent with what would be expected in similar trials or outside trial context. | PN |
| **2.4 If Y/PY to 2.3: Were these deviations likely to have affected the outcome?** |  | NA |
| **2.5. If Y/PY/NI to 2.4: Were these deviations from intended intervention balanced between groups?** |  | NA |
| **2.6 Was an appropriate analysis used to estimate the effect of assignment to intervention?** | “Second, 9 of 409 participants (2%) were missing weight data and 38 (9%) were missing SCL-20 score data at both 6 and 12 months, and were thus excluded from the primary analyses. (p. 878)”  All assigned participants were randomized and only not-eligible participants were excluded. But little information in general about allocation and concealment. | PY |
| **2.7 If N/PN/NI to 2.6: Was there potential for a substantial impact (on the result) of the failure to analyse participants in the group to which they were randomized?** |  | NA |
| **Risk-of-bias judgement** | Low + Low | Low |
| Optional: What is the predicted direction of bias due to deviations from intended interventions? |  | NA |

Domain 3: Missing outcome data

| **Signalling questions** | **Comments** | **Response options** |
| --- | --- | --- |
| **3.1 Were data for this outcome available for all, or nearly all, participants randomized?** | “9 of 409 participants (2%) were missing weight data and ***38 (9%) were missing SCL-20 score data*** at both 6 and 12 months, and were thus excluded from the primary analyses.” (p. 878)  More than 5% of depression severity-data is missing. | N |
| **3.2 If N/PN/NI to 3.1: Is there evidence that the result was not biased by missing outcome data?** | “Because a strict intention-to-treat analysis was not possible, the outcomes for patients who dropped out of the study may not resemble those for patients who remained in the study, and this phenomenon may have resulted in an overestimate of the intervention effects.” (p. 878)  For primary outcomes (including SCL-20): “The analyses used all available data for each outcome, and  missing datawere handled directly through maximum likelihood estimation via mixed modeling. […] The sensitivity analyses using only study measured weights produced consistent results” (p. 873)  The authors cannot exclude bias and mention this as limitation. But they conducted a sensitivity analyses and used ML-estimation that showed little change in results. | PY |
| **3.3 If N/PN to 3.2: Could missingness in the outcome depend on its true value?** |  | NA |
| **3.4 If Y/PY/NI to 3.3: Is it likely that missingness in the outcome depended on its true value?** |  | NA |
| **Risk-of-bias judgement** |  | Low |
| Optional: What is the predicted direction of bias due to missing outcome data? |  | NA |

Domain 4: Risk of bias in measurement of the outcome

| **Signalling questions** | **Comments** | **Response options** |
| --- | --- | --- |
| **4.1 Was the method of measuring the outcome inappropriate?** | SCL-20 = valid instrument for depression severity | N |
| **4.2 Could measurement or ascertainment of the outcome have differed between intervention groups?** | Use of same instrument for both groups and all assessments (baseline, mid- and post-intervention, FU 6 and 12 months) | N |
| **4.3 If N/PN/NI to 4.1 and 4.2: Were outcome assessors aware of the intervention received by study participants?** | Trial protocol: “By design, treatment assignments are identifiable to participants and the lifestyle coach, but the investigators, data and safety monitoring board members, outcome assessors, and data analyst will be blinded throughout the trial. Further, the lifestyle coach will be masked to all outcome measures obtained by outcome assessors during blind assessments.” (p. 4-5) | N |
| **4.4 If Y/PY/NI to 4.3: Could assessment of the outcome have been influenced by knowledge of intervention received?** |  | N |
| **4.5 If Y/PY/NI to 4.4:** **Is it likely that assessment of the outcome was influenced by knowledge of intervention received?** |  | N |
| **Risk-of-bias judgement** |  | Low |
| Optional: What is the predicted direction of bias in measurement of the outcome? |  | NA |

Domain 5: Risk of bias in selection of the reported result

| **Signalling questions** | **Comments** | **Response options** |
| --- | --- | --- |
| **5.1 Were the data that produced this result analysed in accordance with a pre-specified analysis plan that was finalized before unblinded outcome data were available for analysis?** | No changes in analyses-plan from trial protocol | Y |
| **Is the numerical result being assessed likely to have been selected, on the basis of the results, from...** |  |  |
| **5.2. ... multiple eligible outcome measurements (e.g. scales, definitions, time points) within the outcome domain?** | SCL-20 for primary outcome was defined pre intervention and consistently used for assessment and measurements. All pre-defined outcome data reported in results section (no selective reporting). | N |
| **5.3 ... multiple eligible analyses of the data?** | All eligible reported results for outcome measurements correspond to all intended analyses (repeated-measures mixed-effects linear models/generalized linear mixed models). | N |
| **Risk-of-bias judgement** |  | Low |
| Optional: What is the predicted direction of bias due to selection of the reported result? |  | NA |

Overall risk of bias

| **Risk-of-bias judgement** |  | **Low** |
| --- | --- | --- |
| Optional: What is the overall predicted direction of bias for this outcome? |  | NA |

| **Study 2**  **----------------------**  **Study details**   \| **Reference** \| Lv N, Ajilore OA, Xiao L, et al. Mediating Effects of Neural Targets on Depression, Weight, and Anxiety Outcomes of an Integrated Collaborative Care Intervention: The ENGAGE-2 Mechanistic Pilot Randomized Clinical Trial. *Biol Psychiatry Glob Open Sci* 2023;3(3):430–42. doi:10.1016/j.bpsgos.2022.03.012. \| \| --- \| --- \|   **Study design**   \| X \| Individually-randomized parallel-group trial \| \| --- \| --- \|   **For the purposes of this assessment, the interventions being compared are defined as**   \| Experimental: \| Exposure \| Comparator: \| TAU \| \| --- \| --- \| --- \| --- \|  \| **Specify which outcome is being assessed for risk of bias** \| Depressive symptoms severity (SCL-20) \| \| --- \| --- \|  \| **Specify the numerical result being assessed.** \| At 6 months, intervention participants had significantly greater improvements in SCL-20 scores than usual care participants. The between-group mean difference was 20.3 (95% CI = 20.6 to 20.1, p = .002) for SCL-20. \| \| --- \| --- \|   **Is the review team’s aim for this result…?**   \| X \| to assess the effect of *assignment to intervention* (the ‘intention-to-treat’ effect) \| \| --- \| --- \| \| □ \| to assess the effect of *adhering to intervention* (the ‘per-protocol’ effect) \|   **Which of the following sources were obtained to help inform the risk-of-bias assessment? (tick as many as apply)**  X Journal article(s) with results of the trial  X Trial protocol |
| --- | --- | --- | --- | --- | --- | --- | --- | --- | --- | --- | --- | --- | --- | --- | --- | --- |

**Domain 1: Risk of bias arising from the randomization process**

| **Signalling questions** | **Comments** | **Response options** |
| --- | --- | --- |
| **1.1 Was the allocation sequence random?** | “Participants (N = 106) were randomly assigned in a 2:1 ratio to receive the I-CARE2 intervention or usual care using a validated online system (15) based on covariate-adaptive minimization” (p. 432). | Y |
| **1.2 Was the allocation sequence concealed until participants were enrolled and assigned to interventions?** | “Investigators, the Data and Safety Monitoring Board, outcome assessors, and the data analyst were blinded to participants’ treatment assignment until after completing the primary data lock.” (p. 432) | Y |
| **1.3 Did baseline differences between intervention groups suggest a problem with the randomization process?** | “Participants (N = 106) were randomly assigned in a 2:1 ratio to receive the I-CARE2 intervention or usual care using a validated online system (15) based on covariate-adaptive minimization (16). The 2:1 allocation allowed more participants to receive the study intervention (8) without substantially reducing statistical power (17). The minimization method was used to achieve better-than-chance marginal balance across multiple baseline characteristics: age, sex, race/ethnicity, education, BMI, Depression Symptom Checklist-20 (SCL-20) score, and current use of antidepressant medication (yes/no). An imbalance in these covariates between the treatment groups could bias treatment effect estimates.” (p. 432).  No statistical tests of potential differences between groups, but a visual inspection of baseline sociodemographic characteristics did not suggest differences and problems with randomization process. The authors used methods to correct potential imbalances using a study weight (“total imbalance score”). The covariate adaptive minimization method of Pocock and Simon was used to achieve **better-than-chance marginal balance across the baseline characteristics** of clinic, age, sex, race/ethnicity, education level, BMI, SCL-20 score, use of antidepressant medications, and number of hospitalizations during the past year. | PN |
| **Risk-of-bias judgement** |  | Low |
| Optional: What is the predicted direction of bias arising from the randomization process? |  | NA |

**D**omain 2: Risk of bias due to deviations from the intended interventions (effect of *assignment to intervention*)

| **Signalling questions** | **Comments** | **Response options** |
| --- | --- | --- |
| **2.1. Were participants aware of their assigned intervention during the trial?** | No blinding possible because of intervention design | Y |
| **2.2. Were carers and people delivering the interventions aware of participants' assigned intervention during the trial?** |  | Y |
| **2.3. If Y/PY/NI to 2.1 or 2.2: Were there deviations from the intended intervention that arose because of the trial context?** | “Also, an indicator of whether a participant’s outcome was assessed before or after the COVID-19 lockdown date on March 16, 2020, in Illinois (Supplemental Methods and Figure S1) was added as a fixed effect.” p. 432  “Post hoc analysis suggested a trend of greater improvements in SCL-20 and GAD-7 scores, but less weight loss, in the intervention versus usual care after the COVID-19 lockdown (Table S2).” p. 433  “Finally, several changes had to be made in the conduct of the study due to the pandemic (see Supplemental Methods), which may have confounded the results.” P. 440  Supplement:  “3. Changes in Response to the COVID-19 Pandemic Recruitment and baseline data collection were not impacted by the pandemic. In-person visits at follow-up were suspended on 3/16/2020 and restarted on 7/10/2020; final data collection ended on 8/31/2020 (Figure S2). After 3/16/2020, delivery of the intervention sessions was changed from in-person to phone or Zoom videoconference.”  Changes in intervention delivery were made due to Covid-19-restrictions. | PY |
| **2.4 If Y/PY to 2.3: Were these deviations likely to have affected the outcome?** | “Post hoc analysis suggested a trend of greater improvements in SCL-20 and GAD-7 scores, but less weight loss, in the intervention versus usual care after the COVID-19 lockdown.” p. 433  “The intervention effects on depression and anxiety were consistently favorable across the pre-specified sociodemographic subgroups. These effects persisted, and even improved slightly, in GAD-7 after the COVID-19 lockdown in March 2020. For BMI, the between-group mean difference at 6 months was only half of that observed in RAINBOW (20.6 [95% CI = 20.9 to 20.3]). This effect decreased after the lockdown, reflecting the rampant disruptions to people’s lifestyle routines [e.g., detriments in physical activity, diet, and sleep (35,36)] and deleterious secondary health effects [e.g., worsened mental health (5,35) and prevalent weight gain (3,37,38)] disproportionally in underresourced populations (36) during the pandemic. The effects of the pandemic may have contributed to a diluted intervention effect for weight loss” p. 438  It cannot be ruled out that the Covid-19 pandemic and the associated restrictions had an impact on the SCL-20 outcome. There is at least evidence of changes in GAD-7 and BMI. | PY |
| **2.5. If Y/PY/NI to 2.4: Were these deviations from intended intervention balanced between groups?** | Control condition was TAU = no intervention. Pandemic restrictions applied to all participants. | Y |
| **2.6 Was an appropriate analysis used to estimate the effect of assignment to intervention?** |  | NI |
| **2.7 If N/PN/NI to 2.6: Was there potential for a substantial impact (on the result) of the failure to analyse participants in the group to which they were randomized?** |  | NI |
| **Risk-of-bias judgement** | Some Concerns + High | High |
| Optional: What is the predicted direction of bias due to deviations from intended interventions? |  | NA |

Domain 3: Missing outcome data

| **Signalling questions** | **Comments** | **Response options** |
| --- | --- | --- |
| **3.1 Were data for this outcome available for all, or nearly all, participants randomized?** | FlowChart: 106 participants randomized (IG: n=71 🡪 n=66 SCL-20 post-intervention, n=60 FU) – 93% available data Post-intervention, 85% FU = More than 5% missing data | N |
| **3.2 If N/PN/NI to 3.1: Is there evidence that the result was not biased by missing outcome data?** | “Missing data were handled through maximum-likelihood estimation using mixed modeling. Per protocol, in the case of missing study-measured weight, the closest clinical weight documented in the electronic health record within 3 months of the due date of a missed study visit or the self-reported weight (if no clinical weight) was used.” (p. 433)  No sensitivity analyses to correct for bias were reported. Imputation (ML) is not sufficient to exclude bias. | N |
| **3.3 If N/PN to 3.2: Could missingness in the outcome depend on its true value?** |  | NI |
| **3.4 If Y/PY/NI to 3.3: Is it likely that missingness in the outcome depended on its true value?** |  | NI |
| **Risk-of-bias judgement** |  | High |
| Optional: What is the predicted direction of bias due to missing outcome data? |  | NI |

Domain 4: Risk of bias in measurement of the outcome

| **Signalling questions** | **Comments** | **Response options** |
| --- | --- | --- |
| **4.1 Was the method of measuring the outcome inappropriate?** | SCL-20 = valid instrument for depression severity | N |
| **4.2 Could measurement or ascertainment of the outcome have differed between intervention groups?** | Use of same instrument for both groups and all assessments (baseline, mid- and post-intervention) | N |
| **4.3 If N/PN/NI to 4.1 and 4.2: Were outcome assessors aware of the intervention received by study participants?** | “Investigators, the Data and Safety Monitoring Board, outcome assessors, and the data analyst were blinded to participants’ treatment assignment until after completing the primary data lock.” (p. 432) | N |
| **4.4 If Y/PY/NI to 4.3: Could assessment of the outcome have been influenced by knowledge of intervention received?** |  | NA |
| **4.5 If Y/PY/NI to 4.4: Is it likely that assessment of the outcome was influenced by knowledge of intervention received?** |  | NA |
| **Risk-of-bias judgement** |  | Low |
| Optional: What is the predicted direction of bias in measurement of the outcome? |  | NA |

Domain 5: Risk of bias in selection of the reported result

| **Signalling questions** | **Comments** | **Response options** |
| --- | --- | --- |
| **5.1 Were the data that produced this result analysed in accordance with a pre-specified analysis plan that was finalized before unblinded outcome data were available for analysis?** | “Also, an indicator of whether a participant’s outcome was assessed before or after the COVID-19 lockdown date on March 16, 2020, in Illinois (Supplemental Methods and Figure S1) was added as a fixed effect.” (p. 432)  Pilot study, therefore no pre-registration. The authors mention having an analyses plan. They do not report changes in this plan except one additional fixed effect variable to check for influences of covid-19-pandemic. | PY |
| **Is the numerical result being assessed likely to have been selected, on the basis of the results, from...** |  |  |
| **5.2. ... multiple eligible outcome measurements (e.g. scales, definitions, time points) within the outcome domain?** | SCL-20 for primary outcome was defined pre intervention and consistently used for assessment and measurements. All pre-defined outcome data reported in results section (no selective reporting). | N |
| **5.3 ... multiple eligible analyses of the data?** | All eligible reported results for outcome measurements correspond to all intended analyses (generalized linear mixed models). | N |
| **Risk-of-bias judgement** |  | Low |
| Optional: What is the predicted direction of bias due to selection of the reported result? |  | NA |

Overall risk of bias

| **Risk-of-bias judgement** | The study is judged to be at **high risk of bias** in at least one domain for this result (domain 2 & 3) | **High** |
| --- | --- | --- |
| Optional: What is the overall predicted direction of bias for this outcome? |  | NA |

| **Study 3**  **-----------------------**  **Study details**   \| **Reference** \| Young, Myles D.; Drew, Ryan J.; Kay-Lambkin, Frances; Collins, Clare E.; Callister, Robin; Kelly, Brian J. et al. (2021): Impact of a self-guided, eHealth program targeting weight loss and depression in men: A randomized trial. In: *Journal of consulting and clinical psychology* 89 (8), S. 682–694. DOI: 10.1037/ccp0000671. \| \| --- \| --- \|   **Study design**   \| X \| Individually-randomized parallel-group trial \| \| --- \| --- \|   **For the purposes of this assessment, the interventions being compared are defined as**   \| Experimental: \| Exposure \| Comparator: \| Wait list \| \| --- \| --- \| --- \| --- \|  \| **Specify which outcome is being assessed for risk of bias** \| Depressive symptoms severity (PHQ-9) \| \| --- \| --- \|  \| **Specify the numerical result being assessed.** \| At 3 months (post-intervention, primary endpoint), a significant difference favoring the intervention group was observed for change in depressive symptoms (PHQ-9), −2.4, 95% CI [−3.9, −0.8], p < .01, representing a medium effect size, d = 0.55, 95% CI [0.19, 0.90]. These improvements were maintained at 6 months, −2.4, 95% CI [−4.0, −0.7]. \| \| --- \| --- \|   **Is the review team’s aim for this result…?**   \| X \| to assess the effect of *assignment to intervention* (the ‘intention-to-treat’ effect) \| \| --- \| --- \| \| □ \| to assess the effect of *adhering to intervention* (the ‘per-protocol’ effect) \|   **Which of the following sources were obtained to help inform the risk-of-bias assessment? (tick as many as apply)**  X Journal article(s) with results of the trial  X Trial protocol |
| --- | --- | --- | --- | --- | --- | --- | --- | --- | --- | --- | --- | --- | --- | --- | --- | --- |

**Domain 1: Risk of bias arising from the randomization process**

| **Signalling questions** | **Comments** | **Response options** |
| --- | --- | --- |
| **1.1 Was the allocation sequence random?** | “Randomization was stratified based on participants’ antidepressant medication status, depression severity (PHQ-9: <10/ ≥10), and BMI (<33 kg/m2/≥33 kg/m2). Within each stratum, an independent statistician generated unique allocation sequences (1:1 ratio) using a computer-based random number-producing algorithm. These allocation sequences were stored in a restricted computer folder that was inaccessible to those involved in assessment, allocation, and data entry. Prior to assessments, a research assistant prepacked information for each arm into white, opaque envelopes and ordered these within stratum according to the relevant allocation sequence. This research assistant had no further role in the trial. After completing baseline assessments, participants were directed to a separate room to meet with a study chief investigator who was not involved in the assessments. This investigator reviewed the participant’s baseline data before selecting and opening the next available envelope from the appropriate stratification category. The investigator then provided the participant with details on their group assignment and provided men in the intervention group with their program materials.” (p. 687) | Y |
| **1.2 Was the allocation sequence concealed until participants were enrolled and assigned to interventions?** |  | Y |
| **1.3 Did baseline differences between intervention groups suggest a problem with the randomization process?** | “As seen in Table 2, the two study arms were comparable on all baseline characteristics.” (p. 687-688) | N |
| **Risk-of-bias judgement** |  | Low |
| Optional: What is the predicted direction of bias arising from the randomization process? |  | NA |

Domain 2: Risk of bias due to deviations from the intended interventions (*effect of assignment to intervention*)

| **Signalling questions** | **Comments** | **Response options** |
| --- | --- | --- |
| **2.1. Were participants aware of their assigned intervention during the trial?** | “Our study design did not permit blinding of participants (…)” (p. 691)  No blinding possible because of intervention design (only “assessor-blinded”, p. 684). | Y |
| **2.2. Were carers and people delivering the interventions aware of participants' assigned intervention during the trial?** |  | PY |
| **2.3. If Y/PY/NI to 2.1 or 2.2: Were there deviations from the intended intervention that arose because of the trial context?** | “However, our analysis of participant resources indicated mixed adherence to some program tasks. This is consistent with other eHealth interventions (…)” (p. 691)  The authors reported no deviations from the intended intervention besides mixed adherence to some tasks and components. But they described them as being consistent with research and these deviations are also consistent with what could occur outside trial context. | PN |
| **2.4 If Y/PY to 2.3: Were these deviations likely to have affected the outcome?** |  | NA |
| **2.5. If Y/PY/NI to 2.4: Were these deviations from intended intervention balanced between groups?** |  | NA |
| **2.6 Was an appropriate analysis used to estimate the effect of assignment to intervention?** | “Of the 62 men in the intervention group, 51 completed the process evaluation questionnaire at post-test (82%).” (p. 690)  “Participant retention was 80% at 3 months (n = 100) and 78% at 6 months (n = 98), with 82% of men attending at least one assessment post-randomization.” (p. 687)  According to flowchart and table 3, no eligible participants had to be excluded pre- or post-randomization (only missing data at post-intervention assessment which does not effect assignment). | PY |
| **2.7 If N/PN/NI to 2.6: Was there potential for a substantial impact (on the result) of the failure to analyse participants in the group to which they were randomized?** |  | NA |
| **Risk-of-bias judgement** | Low + low | Low |
| Optional: What is the predicted direction of bias due to deviations from intended interventions? |  | Unpredictable |

Domain 3: Missing outcome data

| **Signalling questions** | **Comments** | **Response options** |
| --- | --- | --- |
| **3.1 Were data for this outcome available for all, or nearly all, participants randomized?** | “Participant retention was 80% at 3 months (n = 100) and 78% at 6 months (n = 98), with 82% of men attending at least one assessment post-randomization.” (p. 687)  High proportion of non-completers. | N |
| **3.2 If N/PN/NI to 3.1: Is there evidence that the result was not biased by missing outcome data?** | “Between-group differences in retention were not significant at 3 months (χ2 = 1.15, df = 1, p = .28) or 6 months (χ2 = 0.03, df = 1, p = .87). Men who did not attend follow-up assessments were not significantly different to those who did attend on any demographic variables or baseline study outcomes (all p > .05).” (p. 687) | Y |
| **3.3 If N/PN to 3.2: Could missingness in the outcome depend on its true value?** |  | NA |
| **3.4 If Y/PY/NI to 3.3: Is it likely that missingness in the outcome depended on its true value?** |  | NA |
| **Risk-of-bias judgement** |  | Low |
| Optional: What is the predicted direction of bias due to missing outcome data? |  | NA |

Domain 4: Risk of bias in measurement of the outcome

| **Signalling questions** | **Comments** | **Response options** |
| --- | --- | --- |
| **4.1 Was the method of measuring the outcome inappropriate?** | PHQ-9 = valid instrument, consistent over all assessments  Two additional valid instruments for depressive symptoms: BDI, MDRS-22 | N |
| **4.2 Could measurement or ascertainment of the outcome have differed between intervention groups?** | Same instruments in both groups | N |
| **4.3 If N/PN/NI to 4.1 and 4.2: Were outcome assessors aware of the intervention received by study participants?** | “assessor-blinded” (p. 684) | N |
| **4.4 If Y/PY/NI to 4.3: Could assessment of the outcome have been influenced by knowledge of intervention received?** |  | NA |
| **4.5 If Y/PY/NI to 4.4: Is it likely that assessment of the outcome was influenced by knowledge of intervention received?** |  | NA |
| **Risk-of-bias judgement** |  | Low |
| Optional: What is the predicted direction of bias in measurement of the outcome? |  | NA |

Domain 5: Risk of bias in selection of the reported result

| **Signalling questions** | **Comments** | **Response options** |
| --- | --- | --- |
| **5.1 Were the data that produced this result analysed in accordance with a pre-specified analysis plan that was finalized before unblinded outcome data were available for analysis?** | “(…) the protocol was prospectively registered on the Australia New Zealand Clinical Trials Registry (ACTRN12619001209189).” (p. 684) | PY |
| **Is the numerical result being assessed likely to have been selected, on the basis of the results, from...** |  |  |
| **5.2. ... multiple eligible outcome measurements (e.g. scales, definitions, time points) within the outcome domain?** | PHQ-9 for primary outcome was defined pre intervention and consistently used for assessment and measurements. All pre-defined outcome data reported in results section (no selective reporting). | N |
| **5.3 ... multiple eligible analyses of the data?** | All eligible reported results for outcome measurements correspond to all intended analyses (linear mixed models examined weight and depressive symptoms (PHQ-9) for the impact of group (intervention vs. control), time (categorical) and the group-by-time interaction). | N |
| **Risk-of-bias judgement** |  | Low |
| Optional: What is the predicted direction of bias due to selection of the reported result? |  | NA |

Overall risk of bias

| **Risk-of-bias judgement** |  | Low |
| --- | --- | --- |
| Optional: What is the overall predicted direction of bias for this outcome? |  | NA |
